# Transcriptome profiling reveals links between ParS/ParR, MexEF-OprN, and quorum sensing in the regulation of adaptation and virulence in *Pseudomonas aeruginosa*

**DOI:** 10.1186/1471-2164-14-618

**Published:** 2013-09-13

**Authors:** Dongping Wang, Candace Seeve, Leland S Pierson, Elizabeth A Pierson

**Affiliations:** 1 Department of Plant Pathology and Microbiology, Texas A&M University, College Station, TX 77843-2132, USA; 2 Department of Horticultural Sciences, Texas A&M University, College Station, TX 77843-2133, USA; 3 Department of Biology, Baylor University, Waco, TX 76798, USA

**Keywords:** *Pseudomonas*, Two component signal transduction, *parS/parR*, *mexEF-oprN*, Quorum sensing

## Abstract

**Background:**

The ParS/ParR two component regulatory system plays critical roles for multidrug resistance in *Pseudomonas aeruginosa*. It was demonstrated that in the presence of antimicrobials, ParR enhances bacterial survival by distinct mechanisms including activation of the *mexXY* efflux genes, enhancement of lipopolysaccharide modification through the *arn* operon, and reduction of the expression of *oprD* porin.

**Results:**

In this study, we report on transcriptomic analyses of *P. aeruginosa* PAO1 wild type and *parS* and *parR* mutants growing in a defined minimal medium. Our transcriptomic analysis provides the first estimates of transcript abundance for the 5570 coding genes in *P. aeruginosa* PAO1. Comparative transcriptomics of *P. aeruginosa* PAO1 and *par* mutants identified a total of 464 genes regulated by ParS and ParR. Results also showed that mutations in the *parS/parR* system abolished expression of the *mexEF-oprN* operon by down-regulating the regulatory gene *mexS*. In addition to the known effects on drug resistance genes, transcript abundances of the quorum sensing genes (*rhlIR* and *pqsABCDE-phnAB*) were higher in both *parS* and *parR* mutants. In accordance with these results, a significant portion of the ParS/ParR regulated genes belonged to the MexEF-OprN and quorum sensing regulons. Deletion of the *par* genes also led to increased phenazine production and swarming motility, consistent with the up-regulation of the phenazine and rhamnolipid biosynthetic genes, respectively.

**Conclusion:**

Our results link the ParS/ParR two component signal transduction system to MexEF-OprN and quorum sensing systems in *P. aeruginosa.* These results expand our understanding of the roles of the ParS/ParR system in the regulation of gene expression in *P. aeruginosa*, especially in the absence of antimicrobials*.*

## Background

*Pseudomonas aeruginosa* is a Gram-negative, metabolically versatile and environmentally ubiquitous bacterial species that is capable of surviving in a variety of animal and plant hosts and causing opportunistic infections in humans. It is responsible for serious chronic and often fatal lung infections in patients with cystic fibrosis and acute infections in patients that are immune compromised or have serious burns [1]. Infections caused by *P. aeruginosa* often are difficult to treat due to their intrinsic resistance to diverse antibiotics and their capacity for adaptive resistance [2-4]. In *P. aeruginosa*, major mechanisms of multidrug resistance include the production of enzymes that inactivate β-lactamases and aminoglycosides through modification, alterations in topoisomerases, reduced expression of genes encoding outer membrane proteins such as OprD, and increased expression of genes encoding efflux pumps [5,6]. Additionally *P. aeruginosa* can exhibit adaptive resistance, whereby sub-inhibitory concentrations of antibiotics transiently increase resistance to lethal doses. This adaption occurs largely as a result of expression of the *mexXY*-*oprM* efflux [7] and *arnBCADTEF-ugd* lipopolysaccharide modification operons [8,9].

The versatility of *P. aeruginosa* in adapting to different environments has been attributed in part to the complex regulatory networks that coordinate the control of genes involved in adaptation, including coordination of two-component signal transduction (TCST) systems and quorum sensing [10]. The *P. aeruginosa* genome appears to be especially rich in two-component signal transduction (TCST) systems, which use phosphorylation as a mechanism for responding to specific environmental cues [11]. Annotations of *P. aeruginosa* genomes have identified 123 potential TCSTs, most of which have not been characterized functionally [10]. The ParS/ParR TCST is a key regulatory component for intrinsic and adaptive multidrug resistance in *P. aeruginosa *[8,9]. As is typical of TCST systems, the ParS/ParR system consists of a membrane-bound histidine sensor kinase (ParS) and a cytoplasmic response regulator (ParR). Mutations in *parR* result in susceptibility to a wide range of antibiotics including polymyxin B, gentamycin and tobramycin [8]. Previous microarray analyses identified over 100 genes controlled by the ParS/ParR system *in the presence of antimicrobial agents *[8,9]. Among them are genes encoding the outer membrane porin protein OprD*,* the RND efflux pump MexXY-OprM, and the *arnBCADTEF-ugd* lipopolysaccharide modification operon.

The *P. aeruginosa* genome also contains a diversity of quorum-sensing (QS) systems. QS gene regulation has been described as a method of cell-cell communication used by bacteria to synchronize gene expression within a population [12]. In *P. aeruginosa,* QS depends on the autoinducer synthases LasI, RhlI and PqsABCDH/PhnAB as well as their cognate transcriptional regulators LasR, RhlR and PqsR (MvfR), respectively [13]. LasI and RhlI synthesize the canonical autoinducers 3-oxo-dodecanoyl-homoserine lactone (3-oxo-C_12_-HSL) and butanoyl-homoserine lactone (C_4_-HSL) respectively, which cause transcriptional responses by interacting with LasR and RhlR. In contrast, PqsABCDH/PhnAB catalyzes the synthesis of 2-heptyl-3-hydroxy-4-quinolone (PQS), which in turn regulates gene expression through the PqsR protein [14]. The three QS systems function in a hierarchical manner whereby the LasR/I system positively regulates the RhlR/I system, and PQS is considered the terminal signal [15]. This interlinked QS network controls the expression of multiple virulence factors including exoenzymes, toxins, and secondary metabolites (e.g. chitinase, elastase, protease, exotoxin A, hydrogen cyanide, phenazines, pyoverdin, rhamnolipid) as well as the ability to form biofilms [16]. Indeed, activation of the QS signaling systems in *P. aeruginosa* causes significant transcriptional changes. For instance, transcriptome analysis using microarrays identified 315 QS-induced and 38 QS-repressed genes, representing about 6% of the *P. aeruginosa* genome [17].

RND efflux systems (such as MexXY-OprM, MexAB-OprM, MexCD-OprJ, MexEF-OprN) are important not only for intrinsic and/or adaptive resistance to antimicrobial compounds in *P. aeruginosa,* but they affect the transport of QS signals and precursors and thus QS-dependent phenotypes [18]. Each RND efflux system typically consists of a cytoplasmic membrane component that functions as a transporter (e.g. MexY), an outer membrane component presumed to form channels (e.g. OprM), and a protein presumed to link the two membrane proteins (e.g. MexX) [18]. The RND efflux systems differ somewhat in substrate specificities. For example, MexXY-OprM is capable of excreting aminoglycosides and certain unrelated antibiotics (including macrolides and tetracyclines), whereas MexAB-OprM and MexCD-OprJ are responsible for excreting other antibiotics including quinolones and β-lactams [19]. The MexEF-OprN RND efflux pump transports fluoroquinolones, trimethoprim, as well as chloramphenicol [20,21]. In addition to its role in resistance to antibiotics, the MexAB-OprM efflux pump has been shown to play a role in the selective transport of quorum sensing signals [22,23]. The MexEF-OprN efflux pump also exports the PQS precursor 4-hydroxy-2-heptylquinoline (HHQ) and affects many QS-dependent virulence phenotypes [24]. Indeed, 40% of the genes (102 out of 254) regulated by MexEF-OprN belong to the QS regulon [25].

To date most of what is known about the linkage between TCST regulation, especially via the ParS/ParR operon, quorum sensing, and RND efflux pumps in the control of adaptation and virulence traits comes from studies comparing mutants to wild-type *in the presence of antimicrobials*. Interestingly, quantitative reverse transcriptase polymerase chain reaction (qRT-PCR) analysis revealed that ParR-dependent genes such as *arnBCADTEF-ugd*, *pmrB*, *pagL* and PA1797 are not induced by ParR *in the absence of indolicidin,* suggesting that the ParS/ParR system regulates gene expression in an environment-dependent manner [8]. The objectives of the current study were to identify, using RNA-seq whole transcriptome analysis, genes differentially regulated by the ParS/ParR system *in the absence of antimicrobials*. We discuss the hierarchical relationship of the regulatory elements and the suites of traits controlled by each.

## Results

### Growth dependent expression of *parS* and *parR* in wild type *P. aeruginosa* PAO1

Transcriptomic analysis revealed that in *P. aeruginosa* PAO1, the *parS* (1287 bp) and *parR* (708 bp) genes are located in a single operon, as indicated by the absence of non-coding nucleotides between the two genes. To determine the cell density at which expression of *parS* and *parR* are optimal for transcriptomic analysis, qRT-PCR was conducted using wild-type PAO1 at six different cell densities. The transcript abundances of *parS* and *parR* were similar to each other over time and the highest values were observed at an OD_600_ of 1.2 (5 × 10^9^ cfu/ml, mid log phase) (Additional file 1: Figure S1A, B). Moreover, the qRT-PCR results showed that the transcript abundance of *parS* and *parR* (at OD_600_ = 1.2) was 3–6 fold higher when grown in AB minimal medium + 2% casamino acids (CAA) as compared to LB medium. Thus, subsequent work was performed using *P. aeruginosa* grown in AB minimal medium + 2% CAA at OD_600_ ~ 1.2.

### Quantitative analysis of the wild type *P. aeruginosa* PAO1 transcriptome

**Figure 1:**
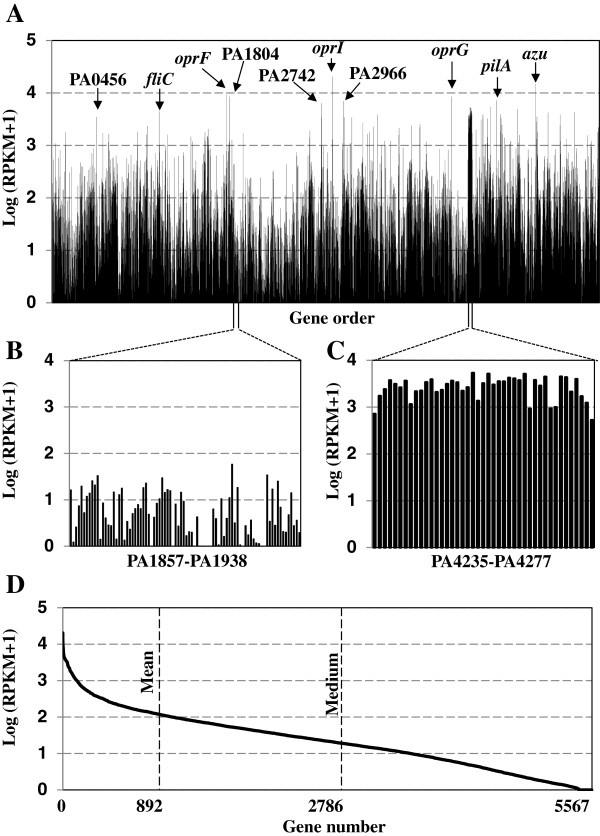
**Quantification of *P. aeruginosa *PAO1 gene expression. A**. Gene expression profile across the entire *P. aeruginosa* PAO1 genome (6.3 Mb). Each column represents one of the 5570 annotated genes in the PAO1 genome, with the *x*-axis showing gene order (from the DNA replication origin), and the *y*-axis showing the log_10_ of transcript abundance (RPKM values) for each gene. The arrows point to a few of the genes or gene clusters that are highly expressed. PA0456: probable cold-shock protein; PA1804: DNA-binding protein HU; PA2742: 50S ribosomal protein L35; PA2966: acyl carrier protein; conserved hypothetical protein; *fliC*: flagellin type B; *pilA*: type 4 fimbrial precursor; *oprF*, *oprG*, *oprI*: outer membrane proteins; *azu*: azurin precursor. **B**. 56.6 kb genome region (PA1857 to PA1938) that shows low levels of gene expression (log (RPKM + 1) < 2). **C**. 33.8 kb genome region (PA4235 to PA4277) that exhibits high levels of gene expression (log (RPKM + 1) > 2). **D**. Distribution of the transcript abundance of the 5570 annotated genes in the PAO1 genome, with the *x*-axis showing gene number (sorted from high to low values), and the *y*-axis showing the log_10_ of transcript abundance for each gene in the wild type. The dashed lines indicate gene numbers where mean and medium values are first detected.

RNA-seq data representing the alignment of sequences (short reads) to coding sequences (CDS) were quantified as reads per kilobase CDS length per million reads analyzed (RPKM), as described previously [26]. The RNA-seq analysis provided a gene expression map showing log (RPKM + 1) values for all 5570 annotated genes in the PAO1 genome (Figure 1A). The mean and medium values were 2.14 and 1.32, respectively, indicting a pronounced skew toward highly expressed genes (Figure 1D). A total of 892 genes were expressed higher than 2.14, whereas the remaining 4678 genes were expressed at lower levels than the mean value (Figure 1D). Genes within large genomic loci (40–90 genes) showed similar patterns in transcript abundance. For example, genes within the region between PA1857 and PA1938 (abundant in hypothetical proteins) were expressed at a relatively low level compared with the more highly expressed genes in the chromosomal segment from PA4235 TO PA4277 (composed of ribosomal proteins, RNA polymerase and elongation factors) (Figure 1B, C).

The top 20 highly expressed genes (excluding genes encoding ribosomal proteins) are included in Additional file 2: Table S2 and many are indicated in Figure 1A. The gene with the greatest transcript abundance in the wild type strain under our experimental conditions was PA2853 (outer membrane lipoprotein *oprI*), having a log (RPKM + 1) value of 4.3 (Figure 1A). Two other membrane protein genes (*oprF* and *oprG*) also had high transcript levels [log (RPKM + 1) values of 3.97 and 3.93, respectively] (Figure 1A). Consistently, the protein levels of OprI, OprF and OprG were shown previously to be highly abundant in *P. aeruginosa*[27,28].

Genes in the top twenty include virulence-related genes such as *pilA *[29], *fliC*[30], *azu *[31], *oprF *[32], *fabF *[33], *capB *[33], *lon *[33] and *sodB *[34]. In contrast, the transcripts of 139 genes were not detected [log (RPKM + 1) = 0] suggesting these genes are barely expressed under the conditions tested. As expected, among the genes with RPKM + 1 values of 0 were 110 genes annotated as encoding hypothetical proteins or proteins with unknown functions.

The relative transcript abundance of the 123 genes annotated as being part of TCST systems (e.g. 64 putative sensor and 59 putative regulator genes including *parS* and *parR)* are provided in Additional file 3: Table S3. The average of the log (RPKM + 1) values for the TCST genes was 1.37, which is relatively low compared to the mean value of 2.14 for all genes. In fact, none of the TCST gene had a log (RPKM + 1) value above 3. The *parS* and *parR* genes were expressed at medium levels [e.g. log (RPKM + 1) values of 1.46 and 1.32, respectively]. Overall, response regulator genes were expressed at higher levels compared with the sensor kinase genes. For instance, among the top twenty highly expressed TCST system genes [log (RPKM + 1) > 1.97], sixteen were response regulator genes. In contrast, sixteen sensor kinase genes were found among the twenty least expressed TCST system genes under these growth conditions [log (RPKM + 1) < 0.8].

### ParS and ParR regulated genes

**Figure 2:**
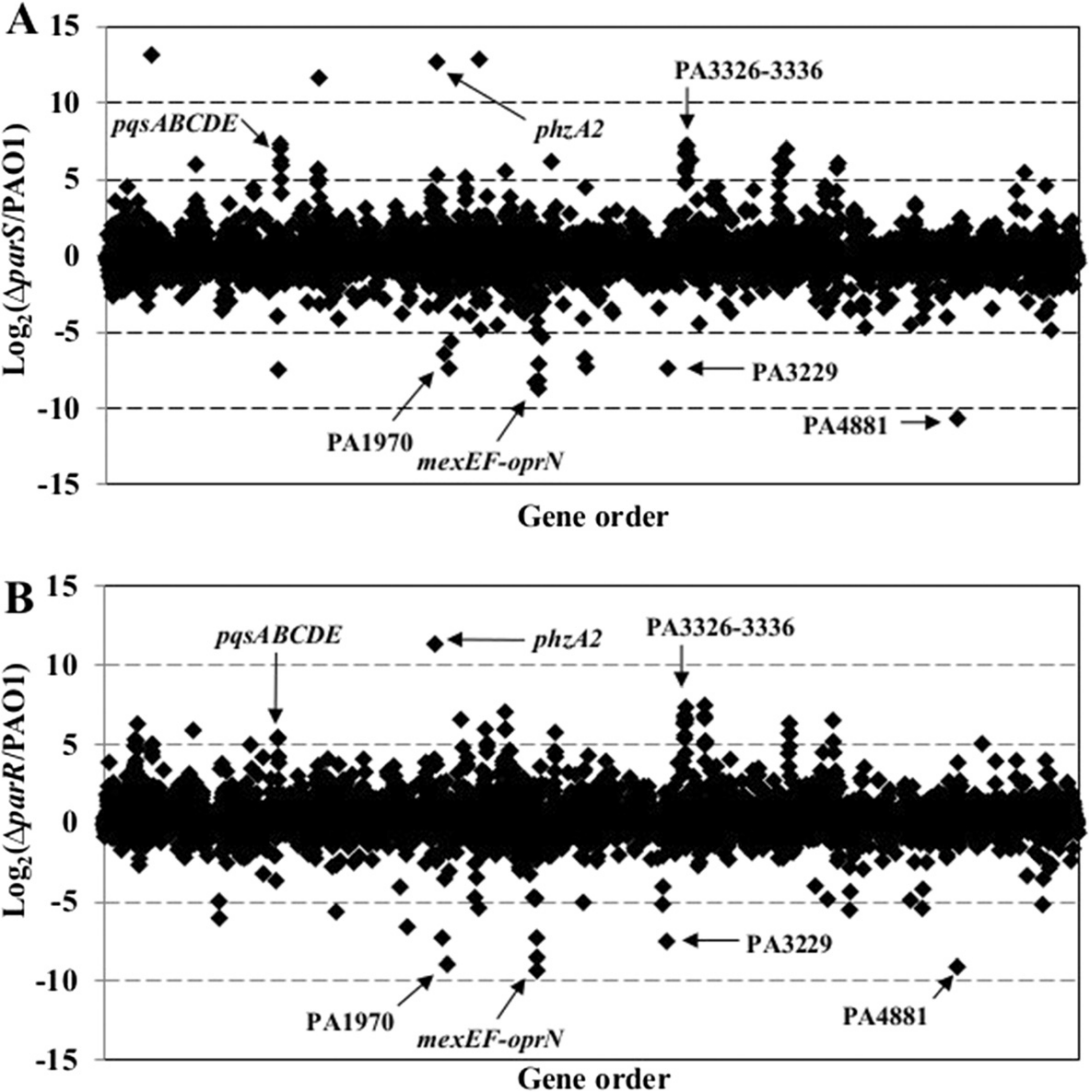
**Differential gene expression profile between wild-type *P. aeruginosa *PAO1 and a *parS *(A) and a *parR *(B) mutant.** Each point represents one of the 5570 annotated genes in the PAO1 genome, with the *x*-axis showing gene order (from the DNA replication origin), and the *y*-axis showing the log_2_ of transcript abundance for each gene in the *parS* or *parR* mutant relative to the wild-type (WT) strain. The arrows point to a few genes or gene clusters that are differentially expressed in both *parS* and *parR* mutants. *pqsABCDE*: PQS signal biosynthetic genes. *phzA2*: phenazine biosynthetic gene; *mexEF-oprN*: RND efflux pump operon; PA1970, PA3229, PA4881: hypothetical proteins; PA3326-PA3336: A 16.6 kb region including *clpP2* (ATP-dependent Clp protease), *fabH2* (3-oxoacyl-[acyl-carrier-protein] synthase III) and many hypothetical genes.

In order to identify genes regulated by the ParS/ParR system in PAO1, mean RPKM values for both *parS* and *parR* mutants were compared with the wild type. The ratios of RPKM values (mutant to wild type) were log-transformed to better illustrate genes that were differentially expressed in *parS* and *parR* mutants. As shown in Figure 2, mutations in *parS* or *parR* caused similar changes in the PAO1 transcriptome at the mid log phase. The transcript abundance of a total of 257 and 331 genes were changed in the *parS* and *parR* mutants compared to wild type, respectively (Additional file 4: Table S4). Of these genes, 124 genes were differentially expressed in both *parS* and *parR* mutants (e.g. the transcript abundance of 100 and 24 genes were higher or lower than wild type, respectively) (Additional file 4: Table S4). These results suggest that mutations in *parS* and *parR* have both common and differential influences on the bacterial transcriptome.

Among the 24 genes activated by both ParS and ParR, only 4 genes (*mexE, mexF, mexS* and *oprN*) have been functionally characterized in *P. aeruginosa* (Table 1) and further discussion of these genes appears below. Among the 100 genes repressed by both ParS and ParR were genes encoding enzymes (e.g. chitinase, elastase, and protease), genes involved in secondary metabolite biosynthesis (e.g. hydrogen cyanide, phenazine, and rhamnolipid synthesis), and genes involved nitrous oxide reduction (Table 1). To validate the expression profiles obtained by RNA-Seq, qRT-PCR was performed on 18 genes; these included genes encoding for components of the quorum sensing systems, enzymes involved in chitin degradation, phenazine and rhamnolipid synthesis, and proteins previously shown to respond to toxic compounds. The data (e.g. fold differences in mutant versus wild type transcript abundances) from the qRT-PCR analysis were comparable to those obtained by the RNA-seq analysis for all selected genes (Additional file 5: Figure S2), thus verifying the RNA-seq data.

**Table 1:** **Selected differentially regulated genes in the *parS *and *parR *mutants compared to WT**

**Locus**	**Gene**	**Log fold**^**ab **^**∆ *parS */WT**	**Log fold**^**ab **^**∆ *parR */WT**	**Protein description**			
**Down-regulated genes**							
PA2491	*mexS*	−3.51	−2.39	Oxidoreductase			
PA2493	*mexE*	−8.21	−7.30	Multidrug efflux membrane fusion protein			
PA2494	*mexF*	−8.75	−9.36	Multidrug efflux transporter			
PA2495	*oprN*	−7.11	−8.53	Multidrug efflux outer membrane protein			
PA2811	*…*	−2.05	**−0.45**	Probable permease of ABC transporter			
PA2812	*…*	−2.62	−0.86	Probable ABC transporter			
PA2813	*…*	−2.44	−1.36	Probable glutathione S-transferase			
PA3229	*…*	−7.4	−5.2	Hypothetical protein			
PA4354	*…*	−3.07	−1.47	Conserved hypothetical protein			
PA4356	*…*	−3.55	−2.94	Xenobiotic reductase			
PA4623	*…*	−4.55	−4.91	Hypothetical protein			
PA4661	*pagL*	−1.01	−0.72	Lipid A 3-O-deacylase			
PA4881	*…*	−10.68	−9.13	Hypothetical protein			
**Up-regulated genes**							
PA0051	*phzH*	3.56	1.40	Phenazine-modifying enzyme			
PA0441	*…*	1.86	1.53	Dihydropyrimidinase			
PA0523	*norC*	3.61	2.52	Nitric-oxide reductase subunit C			
PA0524	*norB*	3.24	1.84	Nitric-oxide reductase subunit B			
PA0525	*norD*	2.56	1.32	Probable dinitrification protein D			
PA1130	*rhlC*	3.07	2.42	Rhamnosyltransferase 2			
PA1671	*stk1*	2.78	2.99	Serine-threonine kinase			
PA1707	*pcrH*	2.09	2.23	Regulatory protein			
PA1778	*cobA*	1.92	2.14	Methyltransferase			
PA2193	*hcnA*	3.86	5.95	Hydrogen cyanide synthase			
PA2300	*chiC*	5.52	2.27	Chitinase			
PA2303	*ambD*	0.92	1.53	Taurine catabolism dioxygenase			
PA1899	*phzA2*	12.66	11.33	Phenazine biosynthesis protein			
PA1900	*phzB2*	5.26	1.79	Phenazine biosynthesis protein			
PA2593	*qteE*	3.18	2.53	Quorum threshold expression element			
PA3326	*clpP2*	2.80	3.33	Protease			
PA3331	*…*	5.82	5.56	Cytochrome P450			
PA3333	*fabH2*	6.82	6.36	3-oxoacyl-[acyl-carrier-protein] synthase III			
PA3478	*rhlB*	3.68	2.50	Rhamnosyltransferase chain B			
PA3479	*rhlA*	4.37	3.31	Rhamnosyltransferase chain A			
PA3724	*lasB*	4.29	3.32	Elastase			
PA3757	*nagR*	1.75	3.48	Transcriptional regulator			
PA3974	*ladS*	1.52	1.40	Sensor protein			
PA4133	*…*	4.07	4.47	Cytochrome C oxidase subunit			
PA4209	*phzM*	4.22	1.94	Phenazine-specific methyltransferase			
PA4211	*phzB1*	5.72	8.13	Phenazine biosynthesis protein			
PA4217	*phzS*	6.04	5.95	Flavin-containing monooxygenase			

^a^Log expression ratio ≥1.0 indicates genes are up-regulated in mutants and ≤ −1 indicates genes are down-regulated in mutants.

^b^The number in bold with P value very close to 0.05. All others P value < 0.05.

### The ParS/ParR system activates the *mexEF-oprN* operon through *mexS*

**Figure 3:**
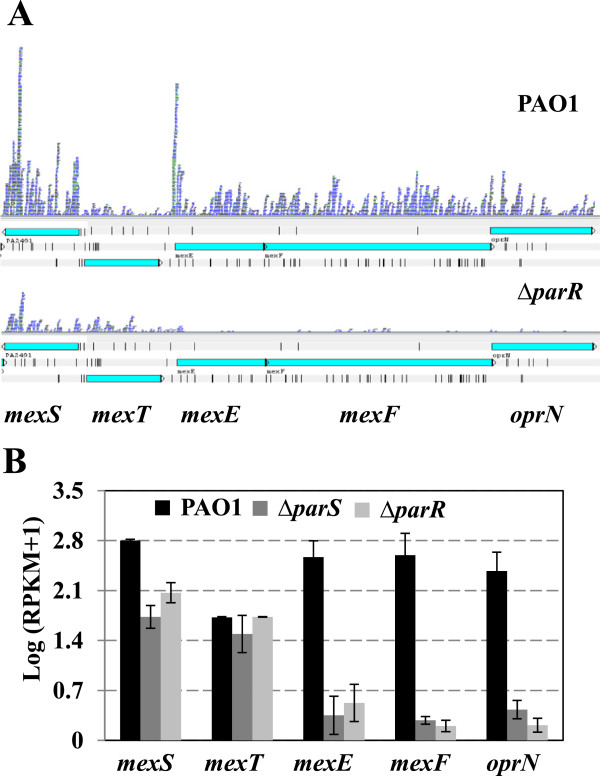
**Expression of *mexT *and *mexEF-oprN *genes in wild type PAO1, *parS *and *parR *mutants. A**. RNA-seq profile showing sequence reads across the *mexT* and *mexEF-oprN* region. RNA-seq results were visualized using the Artemis (http://www.sanger.ac.uk/resources/software/artemis/) genome browser. **B**. Transcript abundance (log_10_ of RPKM values) of *mexS*, *mexT* and *mexEF-oprN* genes in wild type PAO1, *parS* and *parR* mutants. Data points represent means of three replicates ± standard deviations.

The sequence reads from the wild type and *parR* mutant were mapped to the genome sequence of the *mexEF-oprN* region and displayed using Artemis and Bamview (Figure 3A). Compared to the wild type strain, which had abundant reads over the *mexEF-oprN* operon, few reads were detected across this region for the *parR* mutant. The *parS* mutant showed a similar expression pattern to that of the *parR* mutant (data not shown). Consistently, the log (RPKM + 1) values were around 2.5 for *mexE*, *mexF* and *oprN*, for the wild type, whereas deletion of *parS* or *parR* reduced the values to less than 0.8 (Figure 3B). qRT-PCR also confirmed that the expression of *mexF* and *oprN* was highly down-regulated by log (fold changes) of 7.6 ± 0.2 and 6.6 ± 1.8 in the *parR* mutant and by 8.6 ± 1.4 and 7.6 ± 1.6 in the *parS* mutant compared with wild type, consistent with the RNA-seq data (Additional file 6: Figure S3).

As reported previously, expression of the *mexEF-oprN* operon is under the positive control of the DNA-binding protein MvaT [35], the oxidoreductase MexS [36] and the LysR family protein MexT [37]. Transcripts of *mvaT* were expressed at similar high levels in the wild type and mutant strains: log (RPKM + 1) values for wild type, *parS* and *parR* mutants were 3.29 ± 0.04, 3.30 ± 0.15 and 3.35 ± 0.02, respectively. Although the expression of *mexT* was not appreciably altered, mutation in *parS/parR* reduced the transcript levels of *mexS* (Figure 3A, B; Additional file 5: Figure S2). Since regulation of MexEF-OprN by MexS depends on MexT [36], these results suggest that the ParS/ParR system activates the *mexEF-oprN* operon through the MexS-MexT pathway.

### The ParS/ParR system negatively controls quorum sensing

**Figure 4:**
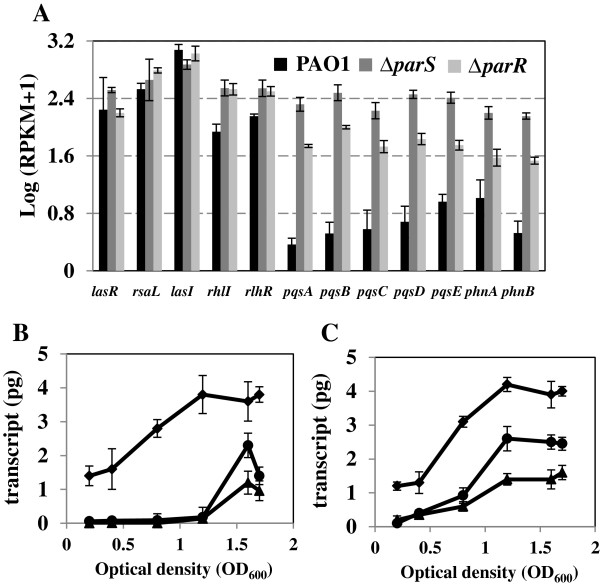
**Impact of the ParS/ParR system on quorum sensing. A**. Transcript abundance (log_10_ of RPKM values) of QS systems in wild type PAO1, *parS* and *parR* mutants. Data points represent means of three replicates ± standard deviations. Expression levels of *lasI, rhlI* and *pqsA* in cultures of wild type PAO1 **(B.)** and Δ*parR***(C.)** at different cell densities. Bacteria were grown in AB minimal medium + 2% casamino acids and RNA was isolated from cells harvested at six different growth stages (OD_600_). The relative abundance of *lasI* (♦), *rhlI* (●) and *pqsA* (▲) was estimated based on *rpoD* transcript quantity in cDNA samples determined by qRT-PCR.

It was shown previously that the MexEF-OprN efflux pump interferes with quorum sensing by extruding HHQ and kynurenine in *P. aeruginosa*[24,25]. Moreover, a quorum sensing regulatory gene encoding the quorum threshold expression element QteE [38] also was differentially expressed in *parS* and *parR* mutants (Table 1). Therefore, the ParS/ParR system has regulatory effects on QS in *P. aeruginosa*. Indeed, transcripts of *rhlI* and *rhlR* were elevated in *parS* and *parR* mutants compared with the wild type (Figure 4A). Although the expression levels of *lasI* and *lasR* were not altered, the *rsaL* gene was slightly increased in the *parR* mutant. The *pqsABCDE* and *phnAB* operons also were expressed at higher levels in the mutant strains. The *pqsABCDE-phnAB* cluster is known to be positively controlled by the cognate regulator PqsR, also named MvfR [39]. Interestingly, *pqsR* was expressed at similar levels in the wild type [log (RPKM + 1) = 2.77 ± 0.15] and mutant strains [log (RPKM + 1) = 2.66 ± 0.21 for ∆*parS* and 2.80 ± 0.13 for ∆*parR*] suggesting that the ParS/ParR system regulates the level of *pqsABCDE*-*phnAB* independently of *pqsR*.

To better understand how ParS-ParR regulates the three QS systems in *P. aeruginosa*, the transcript abundances of *lasI*, *rhlI* and *pqsA* were monitored at six different cell densities in the wild type and *parR* mutant (Figure 4B, C). The *lasI* gene appeared to be constitutively expressed and reached the highest level at an OD_600_ of 1.2 in both wild type and *parR* mutant. In contrast, wild type and the *parR* mutant exhibited different patterns in the expression of the *rhlI* and *pqsA* genes. First, the expression levels of both *rhlI* and *pqsA* were not detectable until cultures reached an OD_600_ of 0.8 in the wild type, whereas these genes were detected at OD_600_ of 0.4 in the mutant strains. Secondly, the transcripts of *rhlI* and *pqsA* genes reached their highest levels at an OD_600_ of 1.6 for the wild type, but at an OD_600_ of 1.2 for the *parR* mutant. Together, these results confirm a role of the ParS/ParR system in controlling the timing of QS gene expression in *P. aeruginosa*.

### Genes known to be activated by ParS/ParR system in the presence of antimicrobials

In the presence of antimicrobial agents such as indolicidin, the ParR protein promotes drug resistance through several known, distinct mechanisms including: activating the *mexXY* efflux genes, suppressing the expression of *oprD* porin, and enhancing lipopolysaccharide modification through the *arn* genes (9). The RNA-seq analysis showed that in the absence of antimicrobials the expression of *oprD* increased by 3.1 and 6.9 fold in *parS* and *parR* mutants, respectively (Table 1), similar to the effect of mutations observed in the presence of antimicrobials. However, the transcript levels of *mexXY-oprM* and *arnBCADTE-ugd* were not appreciably different suggesting that the ParS/ParR system does not have a strong influence on these two operons in defined minimal medium. One possible reason is that the *arnBCADTE-ugd* and *mexXY* operons were expressed at low levels in the wild type strain under our growth conditions. Indeed, the log (RPKM + 1) values of *arnBCADTE-ugd* and *mexXY* were 0.4-1.7 and 0.5-0.7, respectively.

### Negative impact of the ParS/ParR system on phenazine production and motility

**Figure 5:**
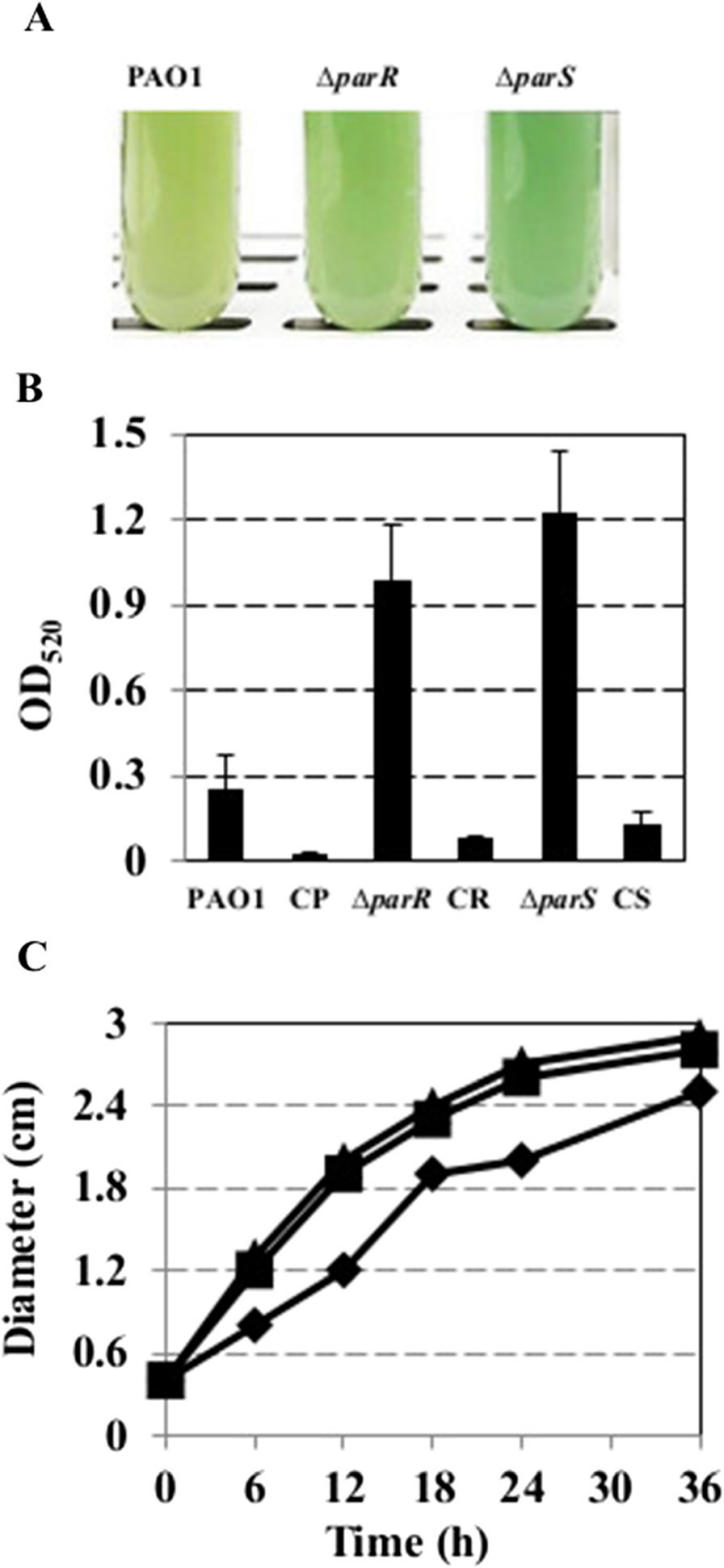
**The ParS/ParR system negatively controls phenazine production and motility. A**. Pyocyanin production of wild-type (PAO1), and mutant strains in AB medium + 2% CAA 24 h post-inoculation. **B**. Pyocyanin production by different strains *in vitro*. Bacterial strains were grown in AB medium +2% CAA for 24 h at 37°C with shaking. Pyocyanin was extracted and quantified at OD_520_. Data points represent the means of three replicates ± standard deviations. Similar results were obtained in at least two independent experiments. CP, CR and CS indicate the wild type, *parR* and *parS* mutants harboring the complementation plasmid pKT2-*parSR*, respectively. **C**. Comparison of motility among wild-type, *parS* and *parR* mutants. Cells were inoculated on the surface of a motility plate containing 0.4% agar as described previously. Plates were incubated at 28°C for 36 h, during which the displacement (diameter) of the outermost edge of the movement was measured. The experiment was performed three times in triplicate. Errors bars were smaller than the symbols.

We noticed previously that the ParS/ParR system controls the production of the phenazine pyocyanin. Especially in pigment-production medium (PPMD), the *parS* and *parR* mutants produce more pyocyanin (green color) than the wild-type (Figure 5A). The impact of the deletion of *parS* or *parR* on phenazine production was quantified using chloroform extraction of cultures grown in PPMD medium followed by spectrophotometric assays as described previously [40]. The amount of phenazine produced by the *parS* and *parR* mutants was greater than two fold the amount produced by the wild-type strain (Figure 5B). Complementation of the mutants by introducing *parS-parR in trans* on a medium-copy-number vector reduced phenazine production below wild type levels, confirming its negative regulatory role in phenazine biosynthesis.

Rhamnolipids are an essential component for *P. aeruginosa* swarming motility [41]. To determine whether the increased expression of rhamnolipid biosynthetic genes (*rhlA*, *rhlB* and *rhlC*) resulted in increased motility in the mutant strains, swarming motility was assessed by inoculating bacterial cells on a motility plate (0.4% agar) and measuring the diameter of the circle covered by bacterial cells for up to 36 h as described previously [42,43]. Both *parS* and *parR* mutants exhibited enhanced motility compared with that of the wild type strain (Figure 5C). These results suggest that the ParS/ParR system is a negative regulator of bacterial motility in *P. aeruginosa*.

qRT-PCR was used to verify phenazine- and motility-related gene expression in these bacterial strains, including *phzA1*, *phzA2*, *phzM*, *rhlA* and *rhlB*, in AB medium + 2% CAA at mid-logarithmic phase. Expression of *phzA1*, *phzA2*, *phzM*, *rhlA* and *rhlB* in the *parS* and *parR* mutants was up-regulated by 2–6 log (fold change), as compared to that of the wild type (Additional file 5: Figure S2). These results further confirmed the regulatory roles of ParS/ParR on phenazine and motility genes.

## Discussion

In this study, we determined the transcript levels of all 5570 of the annotated coding sequences within the *P. aeruginosa* PAO1 genome. To the best of our knowledge, this is the first quantitative transcriptomic atlas of *P. aeruginosa* PAO1. RNA-seq analysis also identified genes regulated by ParS and ParR and linked the ParS/ParR TCST system to the well characterized MexEF-OprN operon and the three quorum sensing systems. This linkage was not identified previously by transcriptomic analyses using microarrays to compare mutants to wild type under different growth conditions (e.g. in the presence of antibiotics). Hence, these results provide important clues toward understanding the complexity of the regulatory roles mediated by ParS/ParR in controlling drug resistance.

Previous microarray studies identified 114 genes controlled by ParR in the presence of 4 μg/ml indolicidin [8] and 17 genes controlled by a point mutation (M59I) in the ParR protein [9]. The two microarray studies shared 14 common genes including *arnABCDEF* and 8 genes (PA1559, PA1660, PA1797, PA2358, PA2655, PA4773, PA4774 and PA4775) encoding hypothetical proteins (Additional file 6: Figure S3A). The agreement between these studies in identifying genes within the *arnBCADTEF-ugd* operon confirms the importance of ParR in the regulation of lipopolysaccharide modification genes in the presence of antimicrobials. Two of those genes *arnF* and PA4773 also were identified as being ParS/ParR regulated in our study (Additional file 4: Table S4). Since *arnF* and PA4773 belong to the *arnBCADTEF-ugd* and PA4473-PA4475 operons, these data indicate that the influence of ParS and ParR on the two operons is weaker in the absence of antimicrobials. Interestingly, data from all three studies indicate that ParS/ParR are responsible for the repression of the *oprD* gene (basic amino acid and carbapenem permeable porin) (Additional file 4: Table S4, Additional file 6: Figure S3A).

The 24 commonly regulated genes identified by our study and the indolicidin treatment studies included 19 genes that were suppressed by ParR in both studies (Table 1; Additional file 4: Table S4). These included are *phzA2, phzB2, phzS* (phenazine biosynthetic genes), *norBCD* (nitric oxide reductase), *chiC* (chitinase), *lecB* (fucose-binding lectin), PA4133 (cytochrome c oxidase) and 10 genes encoding hypothetical proteins. One gene *pagL* encoding a lipid A 3-O-deacylase was down-regulated in both studies. Interestingly, 4 genes including PA0282-83 (sulfate transporter genes), PA4443 (ATP sulfurylase small subunit) and PA4773 (hypothetical protein) that were over-expressed in the *parR* mutant in this study, were down-regulated in the study using indolicidin. These results indicate that induction of a portion of ParR-regulated genes depends on the environmental conditions.

It was reported that the MexEF-OprN efflux pump produces specific transcriptional changes in *P. aeruginosa* regulatory networks [25]. Since both the MexEF-OprN efflux pump and QS systems were regulated by the ParS/ParR system, we compared our transcriptome data with those of the two studies that have contributed to define the MexEF-OprN and QS regulons in *P. aeruginosa*[17,25]. The comparison revealed that approximately 16% and 22% of the genes (74 and 98 out of 464) that were differentially regulated by ParS/ParR belong to the MexEF-OprN and QS regulons, respectively (Additional file 6: Figure S3B). A total of 41 genes were commonly regulated by the three systems (Additional file 7: Table S5). Half of these genes (22) were classified as hypothetical or functionally unknown. Other genes of interest included *pqsABCDE*-*phnAB*, *hcnAC* (hydrogen cyanide synthase), *phzB1* (phenazine biosynthesis), *chiC* (chitinase), *lecB* (fucose-binding lectin), *clpP2* (ATP-dependent protease), *lptF* (lipotoxin), *mexH-opmD* (multidrug efflux pump). Another interesting feature was that all 41 genes were over-expressed in the *parS/parR* mutant. Together, these results suggest that the ParS/ParR regulatory effects are partially mediated by the MexEF-OprN and QS systems.

A total of 33 genes were specifically regulated by ParS/ParR and MexEF-OprN, but not QS (Additional file 6: Figure S3B; Additional file 8: Table S6). Among them, 24 genes were under-expressed in the *parS/parR* mutant including *pagL*, *pncB1* (PA4919, nicotinate phosphoribosyltransferase), *xenB* (PA4356, xenobiotic reductase), *idh* (PA2624, isocitrate dehydrogenase). PncB1 and XenB are enzymes involved in the degradation of nicotinate and trinitrotoluene (TNT), respectively [44,45]. Another interestingly gene cluster was PA2811-PA2813 encoding two ABC transporter and a glutathione S-transferase (GST). GSTs constitute a large family of enzymes that catalyze the addition of glutathione to many toxic exogenous compounds [46]. The 9 genes that were over-expressed included *oprD*, *nosFY* (PA3394-95 nitrous oxide reductases), *hpcB* (PA4124, xenobiotic reductase), *hpaA* (PA4091, 4-hydroxyphenylacetate 3-monooxygenase large chain), PA3951 (molybdopterin biosynthetic protein B1), *narK1* (PA3877, nitrite extrusion protein 1) and PA1875 (probable outer membrane protein). Since the expression of *mexEF-oprN* was activated by ParS/ParR; whereas the expression of *parS* or *parR* was not affected by MexEF-OprN [25], it is reasonable to speculate that the ParS/ParR system functions upstream of MexEF-OprN. These results also suggest that the ParS/ParR system may control membrane permeability and detoxification genes through the positive control of the MexEF-OprN efflux pump, but not through QS. A total of 57 genes were controlled by ParS/ParR and the QS systems, but not the MexEF-OprN operon (Additional file 6: Figure S3B; Additional file 9: Table S7). Consistent with the up-regulation of QS-controlled genes, the expression of *rhlI* and *rhlR* was increased 2–5 fold in *parS* and *parR* mutants. The QS regulatory gene *qteE* was also in this group. Some other noteworthy members of this group were genes encoding RND efflux transporters (PA3676-77 and *triC*), secondary metabolism genes (*rhlABC*, *aprAE* and *lasB*), cytochrome c oxidases (PA0105-08, PA1556) and regulatory genes (PA2591 and PA3347). These results suggest the possibility that the impact of ParS and ParR on QS is partially mediated by the RhlR/I system.

The *ladS* gene encoding a two component sensor protein was expressed at high levels in both *parS* and *parR* mutants compared with the wild type (Table 1). Previous studies showed that the LadS protein activates *P. aeruginosa* QS expression through the GacS/GacA two component system and the regulatory RNA RsmZ [47]. This observation linked the ParS/ParR system with the well-characterized GacS/GacA regulatory system. Another gene, *phzH* encoding a phenazine terminal modifying enzyme, was also negatively regulated by the ParS/ParR system (Table 1). PhzH is a unique transamidase involved in the conversion of phenazine-1-carboxylic acid (PCA) to phenazine-1-carboxamide (PCN) [48]. Unlike *phzM* and *phzS* (phenazine modifying enzymes), which were positively controlled by QS, *phzH* was not regulated by the QS and MexEF-OprN systems [17,25]. Five genes (PA3095, PA3096, PA3099, PA3102 and PA3105) annotated as encoding components of a type II secretion system (T2SS) also were over-expressed (2 to 6-fold) in the *parS* and *parR* mutants relative to the wild type, suggesting positive control by ParS and ParR (Additional file 4: Table S4). The T2SS is a secretory pathway for most extracellular proteins. Although, these genes were not identified as targets for QS by the transcriptomic studies [17], transcriptional fusion assays showed that their expression was influenced by the Rhl and Las QS systems [49].

## Conclusion

**Figure 6:**
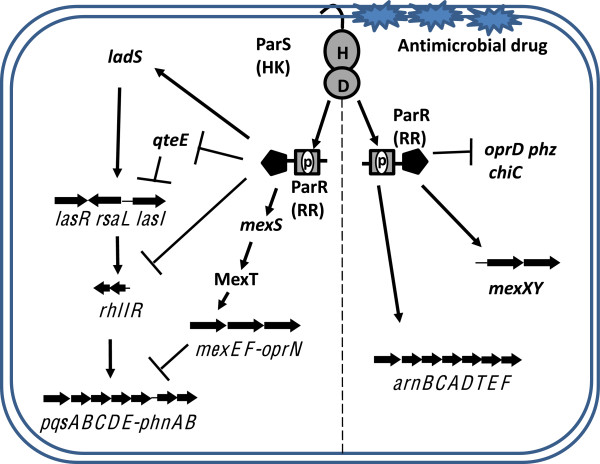
**ParS/ParR hierarchical control in *P. aeruginosa*.** The model shows the predicted position of the ParS/ParR system in relation to other known regulators of QS. ParS is a transmembrane protein according to *in silico* predictions based on amino acid sequences. Amino acids H (histidine) and D (aspartate) involved in phosphorylation are indicated. The solid area of ParR indicates the DNA-binding domain. Solid arrows and blunt lines point to genes (or processes) that are positively or negatively affected, respectively. ParR controls the expression of *mexEF-oprN, qteE* and *ladS*, which in turn regulate QS through different mechanisms. HK: histidine kinase; RR: response regulator; P: phosphoryl group.

Previous microarray studies demonstrated that the ParS/ParR TCST system controls the expression of the lipopolysaccharide modifying (*arnBCADTEF-ugd*), efflux (*mexXY*), porin (*oprD*), chitinase (*chiC*) and phenazine biosynthetic genes (*phzA2B2*) when exposed to subinhibitory concentrations of antimicrobial reagents [8,9]*.* In this study, the major components of the ParS/ParR signal transduction pathway in *P. aeruginosa* PAO1 were identified in bacteria grown without antimicrobials (Figure 6). Significantly, we showed that the three QS regulatory genes, the gene cluster including the *mexEF-oprN* efflux system, the *ladS* sensor kinase and the quorum threshold expression element *qteE* were differentially regulated by the ParS/ParR system. The sensor kinase LadS is an activator of the GacS/GacA two component system and it positively regulates the levels of Las QS system through the titration of the translational regulatory protein RsmA [50]. In contrast, expression of the QteE protein reduces LasR protein stability without affecting its expression [38]. Finally, the MexEF-OprN efflux pump is a transporter of the PQS precursor HHQ and negatively controls the expression of the *pqsABCDE-phnAB* cluster [24,25]. These results indicate that the ParS/ParR system regulates QS at both transcriptional and translational levels through multiple mechanisms. Since the expression of *parS* and *parR* is not controlled by QS or MexEF-OprN, whereas both QS and MexEF-OprN gene are regulated by ParS and ParR, we conclude that the ParS/ParR system is on the top of this hierarchical regulatory cascade.

## Methods

### Bacterial strains and growth conditions

The wild type strain PAO1 and its *parS* and *parR* mutants were obtained from the *P. aeruginosa* PAO1 transposon mutant library [51] (Table 2). Analysis of RNA transcript abundance indicated that the insertions did not cause polar effects since the expression of PA1797, the ORF immediately downstream of *parS* and *parR*, was not affected by either insertion (data not shown). Liquid LB medium, pigment production medium (PPMD) or AB minimal medium supplemented with 2% casamino acids (AB + 2% CAA) (Difco, Becton Dickinson and Company, Franklin Lakes, NJ) were used for culturing *P. aeruginosa* as described previously [52]. The following antibiotics were added to the medium when necessary: ampicillin (Ap) 100 μg ml^-1^, kanamycin (Km) 50 μg ml^-1^, and gentamicin (Gn) 30 μg ml^-1^.

**Table 2:** Bacterial strains and plasmids used in this study

**Strains and plasmids**	**Relevant characters**^**a**^	**Reference or source**
***P. aeruginosa***		
PAO1	wild-type (WT)	[51]
∆*parS*	PAO1 Tn5 mutant, insertion at bp 402 in *parS,* Gn^R^	[51]
∆*parR*	PAO1 Tn5 mutant, insertion at bp 299 in *parR,* Gn^R^	[51]
***E. coli***		
DH5α	F^-^*recA1 endA1 hsdR17 supE44 thi-1 gyrA96 relA1* Δ (*argF-lacZYA*)*I169 ϕ* 80*lacZ*ΔM15λ^-^	GIBCO-BRL
**Plasmids**		
pPROBE-KT2	Km^R^, GFP based promoter trap vector containing a promoter-less *gfp* gene	[58]
pKT2-*parSR*	2.3 kb DNA fragment containing *parS* and *parR* genes in pPROBE-KT2	This study

^a^Km^R^ and Gn^R^ = kanamycin and gentamycin resistance, respectively.

### DNA manipulation and sequence analysis

Standard procedures were used for plasmid isolation, cloning, restriction enzyme digestion and T4 DNA ligation [53]. Polymerase chain reaction (PCR) was carried out using Invitrogen *Taq* DNA polymerase (Life Technologies, Carlsbad, CA) at 95°C for 5 min, followed by 30 cycles of 95°C for 30 sec, 60°C for 30 sec and 72°C for 90 sec, and a final elongation step of 70°C for 10 min. DNA sequencing was performed at the Genome Technology Lab (GTL) within the Texas A&M University Institute for Plant Genomics & Biotechnology.

### RNA preparation

Three biological replicates of every strain were started from single colonies located on three separate plates containing AB + 2% CAA and then transferred to 10 ml AB + 2% CAA broth. All cultures were grown at 37°C with shaking (200 rpm) to an approximate OD_600_ = 1.2. Cell cultures collected at OD_600_ = 1.2 were diluted to OD_600_ = 0.3 with AB + 2% CAA broth. RNA extraction was performed as described previously [54-56] with one exception: contaminating genomic DNA was removed off-column with Turbo DNA-free DNase (Life Technologies, Carlsbad, CA). Elimination of contaminating DNA was confirmed via qPCR amplification of the *rpoD* gene with SYBR green® dye on an ABI 9400HT PCR machine (Life Technologies, Carlsbad, CA). RNA samples were ethanol-precipitated and resuspended in 0.1% diethylpyrocarbonate (DEPC). RNA quantification was performed using an Agilent 2100 Bioanalyzer (Agilent Technologies, Santa Clara, CA) at the Texas A&M GTL.

### RNA-seq analysis

RNA-seq was performed as described previously [26]. Briefly, ribosomal RNA (rRNA) was depleted from ~9 μg of total RNA using the RiboZero rRNA depletion kit (for Gram-negative bacteria, Epicentre Biotechnologies, Madison, WI). Strand-specific cDNA libraries were constructed using the SOLiD Total RNA-Seq kit. Paired-end sequencing was conducted by the University of Texas Genomic Sequencing and Analysis Facility on a Life Technologies SOLiD 5500xl sequencing system with a targeted sequencing depth of six-million paired-end reads per sample. Filtering and alignment of the SOLiD 4 paired-end data was performed at the UTGSAF using the AB SOLiD BioScope Whole Transcriptome pipeline (v1.3), for whole-transcriptome RNA-seq analysis. Mapped reads were visualized using BamView in Artemis 13.2.0 [57].

To determine RNA transcriptional abundance for each gene, the number of reads that mapped within each annotated coding sequence (CDS) was determined. The number of reads per kb of transcript per million mapped reads (RPKM) was used to normalize the raw data [26], and mean RPKM values were determined for the three biological replicates. The complete dataset including raw and processed data has been deposited at the National Center for Biotechnology Information (NCBI), Accession No. GSE44681. Comparisons were performed using a modified t-test [26]. A ratio of the mean RPKM values (mutant/WT) was determined for each gene. Ratios over 2 or below 0.5 and p-value < 0.05 were considered differentially expressed [26].

### qPCR methods and analysis

qPCR was performed at the Texas A&M GTL using a previously described method [26]. RNA was reverse-transcribed using random primers (Invitrogen) and Superscript III (Invitrogen) at 50°C for 1 h and inactivated at 75°C for 15 min. SYBR Green reactions were performed using the ABI 7900 HT Fast System (Applied Biosystems, Foster City, CA) in 384 well optical reaction plates. Aliquots (1 μl) of cDNA (2 ng/reaction) or water (no-template control) were used as template for qPCR reactions with Fast SYBR Green PCR Master Mix (Applied Biosystems) and primers (500 nM final concentration). Primer pairs parSRT1-parSRT2, parRRT1-parRRT2, lasIRT1-lasIRT2, lasRRT1-lasRRT2, rhlIRT1-rhlIRT2, rhlRRT1-rhlRRT2, pqsART1-pqsART2, pqsCRT1-pqsCRT2, pqsDRT1-pqsDRT2, pqsRRT1-pqsRBRT2, qteERT1-qteERT2, mexSRT1-mexSRT2, mexFRT1-mexFRT2, oprNRT1-oprNRT2, chiCRT1-chiCRT2, phzA1RT1-phzA1RT2, phzA2RT1-phzA2RT2, phzMRT1-phzMRT2, rhlART1-rhlART2, rhlBRT1-rhlBRT2 and rpoDRT1-rpoDRT2 were used to detect the expression of *parS*, *parR*, *lasI*, *lasR, rhlI, rhlR, pqsA, pqsC, pqsD, pqsR, qteE, mexS, mexF, oprN, chiC, phzA1*, *phzA2, phzM, rhlA, rhlB* and *rpoD* genes, respectively (Additional file 10: Table S1). qPCR amplifications were carried out at 50°C for 2 min, 95°C for 10 min, followed by 40 cycles of 95°C for 15 sec and 60°C for 1 min, and a final dissociation curve analysis step from 65°C to 95°C. Two technical replicates of each of three biological replicates were used for each experiment. Amplification specificity for each reaction was confirmed by the dissociation curve analysis. Ct values determined by the software were then used for further ∆∆Ct analysis. The *rpoD* gene was used as the reference gene to normalize samples and a relative quantification (RQ) value was calculated for each gene with the control group as a reference [26]. For quantification of transcript abundance, a standard curve was generated using purified *rpoD* PCR product over a dilution range of known concentrations and *rpoD* transcript quantity in cDNA samples determined by quantitative real-time PCR was used to estimate the relative amount of template concentrations of the experimental genes.

### Cloning of the *parR-parS* operon

In order to determine whether complementation of the Δ*parR* and Δ*parS* mutants restored normal phenazine production, the *parR-parS* flanking sequences were used to design primers (par1-par2) to amplify the two genes and their promoter sequence. Following amplification, the PCR product was directly cloned into pTOPO 2.1 (Invitrogen). Transformants were selected on LB plates supplemented with 100 μg ml^-1^ Ap. The pTOPO-*parRS* construct and pKT2 vector [58] were digested by *Eco*RI and *Bam*HI and ligated resulting in plasmid pKT-parRS (Table 2). The plasmid was introduced into *P. aeruginosa* strains by electroporation as described previously [52]. Transformants were selected on LB plates supplemented with 50 μg ml^-1^ Km. To confirm transformation, the genotype was confirmed by both enzymatic digestion and sequencing.

### Quantification of phenazine production

*P. aeruginosa* strains were grown with aeration at 37°C in PPMD for 24 h. Phenazines were extracted and quantified by UV-visible light spectroscopy as described previously [40]. Briefly, phenazines were extracted with chloroform from culture supernatants and then extracted with an equal volume of HCl (0.2 N); optical density was measured at OD_520_ nm. The absorbance for each sample was normalized to the total absorbance of the 10-ml culture.

### Bacterial swarming motility assays

For *P. aeruginosa* PAO1, *parS* and *parR* mutants, bacterial cell suspensions were grown overnight in LB broth. Five μl of the bacterial suspensions were plated onto the center of motility agar plates (10 g tryptone, 5 g NaCl, 3.5 g agar per liter distilled water) as described previously [42,43]. Diameters were determined following incubation at 28°C for 36 h. The experiments were repeated at least three times.

## Competing interests

The authors declare that they have no competing interests.

## Authors’ contributions

Experiments conceived and designed by: EAP LSP DPW CS. Experiments were performed and analyzed by: DPW CS EAP LSP. Contributed reagents/materials/analysis tools: EAP LSP. Wrote the paper: DPW CS LSP EAP. All authors read and approved the final manuscript.

## Supplementary Material

Additional file 1: Figure S1 Transcript abundance of *parS* and *parR* mutants at different cell densities. **A**. Growth of PAO1 in AB minimal medium + 2% CAA assessed as OD_600_ (●) or Log cfu/ml (○). he arrow indicates the time at which cells were harvested for RNA-seq analysis. **B**. PA01 was grown in AB minimal medium + 2% CAA and RNA was isolated from cells harvested at six different growing stages (OD_600_). A standard curve was generated using purified *rpoD* PCR product over a dilution range of known concentrations and the relative abundance of *parS* and *parR* was estimated based on *rpoD* transcript quantity in cDNA samples determined by qRT-PCR. Click here for file

Additional file 2: Table S2 Top twenty highly expressed genes in *P. aeruginosa* PAO1. Click here for file

Additional file 3: Table S3 Transcript levels of the 123 TCST genes in *P. aeruginosa* PAO1. Click here for file

Additional file 4: Table S4 Differentially expressed genes in *parS* and *parR* mutant compared to wild type strain PAO1. Up- and down-regulated genes (ratios) are indicated by red and green, respectively. Genes with p-values < 0.05 are highlighted in yellow. Click here for file

Additional file 5: Figure S2 Validation of RNA-seq results by qRT-PCR. Relative gene expression levels in the *parS* and *parR* mutants compared to the wild type strain. Bacterial strains were grown in 5 mL AB medium + 2% CAA. Relative expression of 16 selected genes, normalized to the expression value of the *rpoD* gene, was determined by qRT-PCR after 16 h growth (OD_600_ at 1.2). Data points represent means ± SD of three replicates. These experiments were repeated at least twice and similar results were obtained. Click here for file

Additional file 6: Figure S3 **A**. Comparison of ParS/ParR-regulated genes with ParR-regulated genes in the presence of 4 μg/ml indolicidin (indicated as *parR1*; [8]) and with genes differentially regulated by a ParR point mutation (indicated as *parR2*; [9]). **B**. Venn diagram comparing the number of genes regulated by the three regulons: ParS/ParR (this study), QS [17] and MexEF-OprN [25]. Click here for file

Additional file 7: Table S5 Genes commonly regulated by ParS/ParR, MexEF-OprN and QS. Click here for file

Additional file 8: Table S6 Genes commonly regulated by ParS/ParR and MexEF-OprN, but not QS. Click here for file

Additional file 9: Table S7 Genes commonly regulated by ParS/ParR and QS, but not MexEF-OprN. Click here for file

Additional file 10: Table S1 Oligonucleotides used for gene cloning and qRT-PCR. Click here for file
