# Supplementary figures and images for: Transcriptome profiling reveals links between ParS/ParR, MexEF-OprN, and quorum sensing in the regulation of adaptation and virulence in Pseudomonas aeruginosa
Source: BMC Genomics. 2013 Sep 13;14:618. doi: 10.1186/1471-2164-14-618 (PMC3848899; doi:10.1186/1471-2164-14-618)

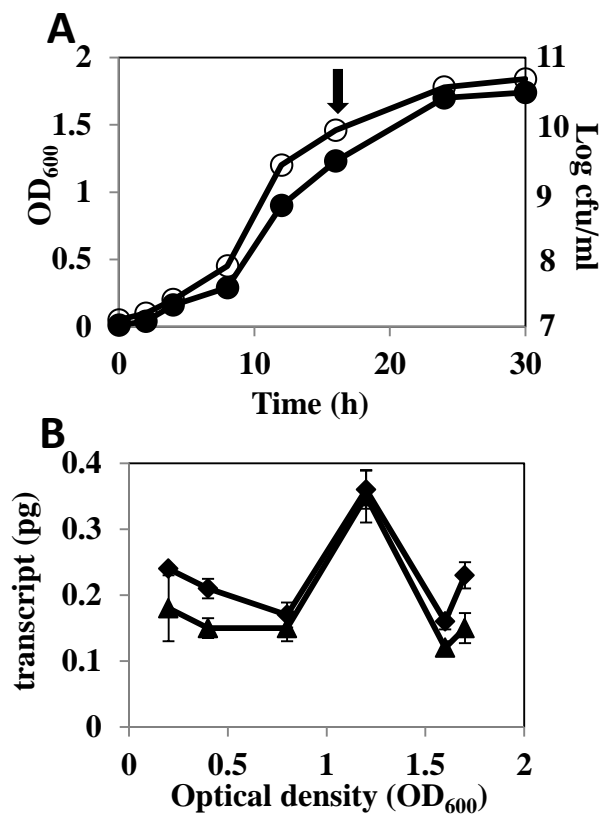

Wang et al., Supplementary Figure 1

Supplement: Additional file 1: Figure S1 — Transcript abundance of parS and parR mutants at different cell densities. A. Growth of PAO1 in AB minimal medium + 2% CAA assessed as OD600 (●) or Log cfu/ml (○). he arrow indicates the time at which cells were harvested for RNA-seq analysis. B. PA01 was grown in AB minimal medium + 2% CAA and RNA was isolated from cells harvested at six different growing stages (OD600). A standard curve was generated using purified rpoD PCR product over a dilution range of known concentrations and the relative abundance of parS and parR was estimated based on rpoD transcript quantity in cDNA samples determined by qRT-PCR. [file 1471-2164-14-618-S1.pdf]

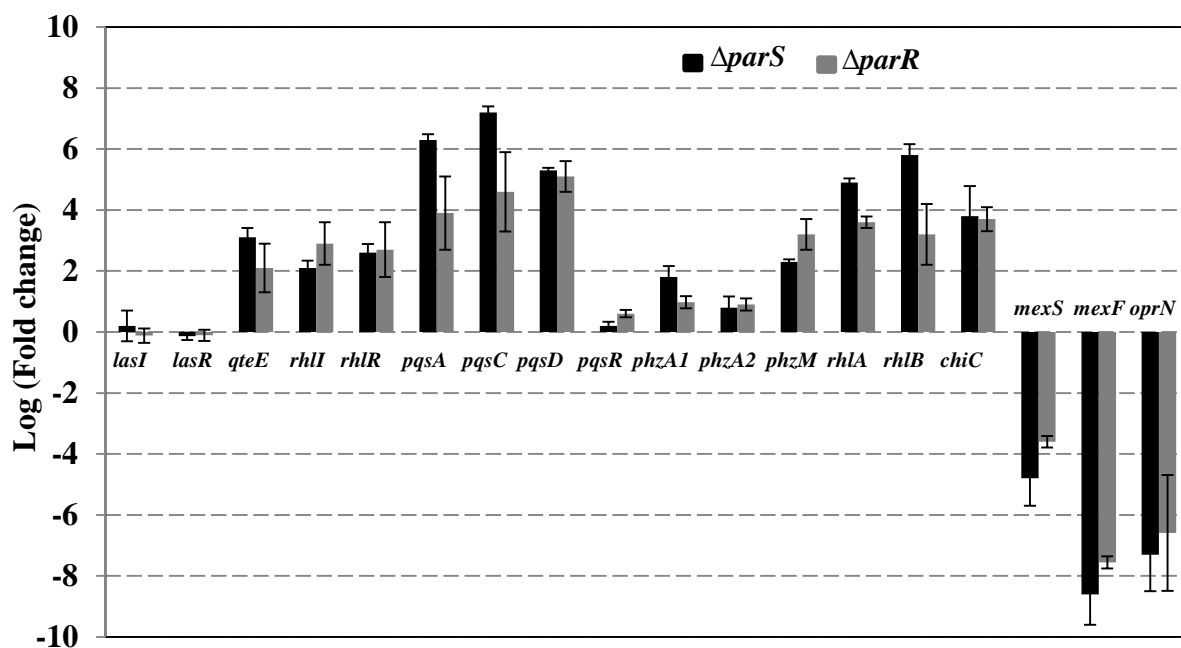

Wang et al., Supplementary Figure 2

Supplement: Additional file 5: Figure S2 — Validation of RNA-seq results by qRT-PCR. Relative gene expression levels in the parS and parR mutants compared to the wild type strain. Bacterial strains were grown in 5 mL AB medium + 2% CAA. Relative expression of 16 selected genes, normalized to the expression value of the rpoD gene, was determined by qRT-PCR after 16 h growth (OD600 at 1.2). Data points represent means ± SD of three replicates. These experiments were repeated at least twice and similar results were obtained. [file 1471-2164-14-618-S5.pdf]

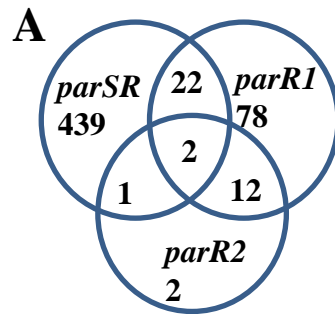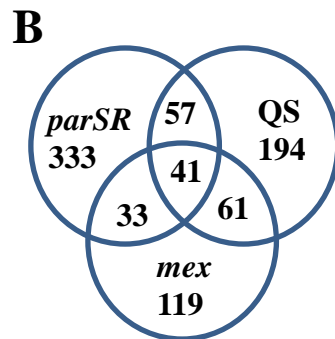

Supplement: Additional file 6: Figure S3 — A. Comparison of ParS/ParR-regulated genes with ParR-regulated genes in the presence of 4 μg/ml indolicidin (indicated as parR1; [8]) and with genes differentially regulated by a ParR point mutation (indicated as parR2; [9]). B. Venn diagram comparing the number of genes regulated by the three regulons: ParS/ParR (this study), QS [17] and MexEF-OprN [25]. [file 1471-2164-14-618-S6.pdf]
