# Supplementary material for: Transcriptome profiling reveals links between ParS/ParR, MexEF-OprN, and quorum sensing in the regulation of adaptation and virulence in Pseudomonas aeruginosa
Source: BMC Genomics. 2013 Sep 13;14:618. doi: 10.1186/1471-2164-14-618 (PMC3848899; doi:10.1186/1471-2164-14-618)
Supplement: Additional file 2: Table S2 — Top twenty highly expressed genes in P. aeruginosa PAO1. [file 1471-2164-14-618-S2.doc]

| Rank | Locus | Gene Name | Description | Gene Size  (bp) | Log(RPKM+1) |
| --- | --- | --- | --- | --- | --- |
| 1 | PA2853 | *oprI* | outer membrane lipoprotein precursor | 252 | 4.31 |
| 2 | PA4922 | *azu* | azurin precursor | 447 | 4.17 |
| 3 | PA1777 | *oprF* | major porin | 1053 | 3.97 |
| 4 | PA4067 | *oprG* | outer membrane protein precursor | 699 | 3.93 |
| 5 | PA1804 | *lon* | DNA-binding protein HU | 273 | 3.93 |
| 6 | PA4525 | *pilA* | type 4 fimbrial precursor | 450 | 3.85 |
| 7 | PA2966 | *fabF* | acyl carrier protein | 237 | 3.84 |
| 8 | PA4463 | *…* | sigma factor modulation protein | 309 | 3.74 |
| 9 | PA1092 | *fliC* | flagellin | 1467 | 3.65 |
| 10 | PA5172 | *argF* | ornithine carbamoyltransferase | 1011 | 3.59 |
| 11 | PA4385 | *groL* | chaperonin | 1644 | 3.59 |
| 12 | PA3309 | *…* | conserved hypothetical protein | 456 | 3.58 |
| 13 | PA4238 | *rpoA* | RNA polymerase alpha chain | 1002 | 3.58 |
| 14 | PA0456 | *…* | probable cold-shock protein | 210 | 3.54 |
| 15 | PA4386 | *groS* | chaperonin | 294 | 3.53 |
| 16 | PA4366 | *sodB* | superoxide dismutase | 582 | 3.49 |
| 17 | PA4578 | *…* | hypothetical protein | 489 | 3.39 |
| 18 | PA5171 | *arcA* | arginine deiminase | 1257 | 3.38 |
| 19 | PA0973 | *oprL* | peptidoglycan associated lipoprotein | 507 | 3.37 |
| 20 | PA3266 | *capB* | cold acclimation protein B | 210 | 3.35 |
