# Supplementary material for: Transcriptome profiling reveals links between ParS/ParR, MexEF-OprN, and quorum sensing in the regulation of adaptation and virulence in Pseudomonas aeruginosa
Source: BMC Genomics. 2013 Sep 13;14:618. doi: 10.1186/1471-2164-14-618 (PMC3848899; doi:10.1186/1471-2164-14-618)
Supplement: Additional file 3: Table S3 — Transcript levels of the 123 TCST genes in P. aeruginosa PAO1. [file 1471-2164-14-618-S3.doc]

| Locus | Description | Gene Size | Log(RPKM+1) |
| --- | --- | --- | --- |
| PA0408 | twitching motility protein PilG | 408 | 2.85 |
| PA0409 | twitching motility protein PilH | 366 | 2.73 |
| PA4843 | probable two-component response regulator | 1629 | 2.72 |
| PA4493 | RoxR | 561 | 2.49 |
| PA0179 | probable two-component response regulator | 366 | 2.48 |
| PA5261 | alginate biosynthesis regulatory protein AlgR | 747 | 2.46 |
| PA1456 | two-component response regulator CheY | 375 | 2.43 |
| PA1179 | two-component response regulator PhoP | 678 | 2.39 |
| PA2586 | response regulator GacA | 645 | 2.33 |
| PA4494 | RoxS | 1269 | 2.32 |
| PA3879 | two-component response regulator NarL | 660 | 2.27 |
| PA4726 | two-component response regulator CbrB | 1437 | 2.25 |
| PA2798 | probable two-component response regulator | 1185 | 2.24 |
| PA1458 | probable two-component sensor | 2262 | 2.24 |
| PA0413 | chemotactic signal transduction system | 7419 | 2.13 |
| PA5200 | AmgR | 744 | 2.11 |
| PA5364 | probable two-component response regulator | 903 | 2.03 |
| PA5262 | alginate biosynthesis protein AlgZ/FimS | 1077 | 2.01 |
| PA0601 | probable two-component response regulator | 633 | 1.97 |
| PA0178 | probable two-component sensor | 1920 | 1.97 |
| PA4296 | two-component response regulator, PprB | 828 | 1.97 |
| PA3948 | Two-component response regulator RocA1 | 630 | 1.95 |
| PA3206 | probable two-component sensor | 1338 | 1.92 |
| PA2572 | probable two-component response regulator | 1344 | 1.87 |
| PA3192 | two-component response regulator GltR | 729 | 1.86 |
| PA2824 | probable sensor/response regulator hybrid | 2361 | 1.80 |
| PA4856 | RetS (Regulator of Exopolysaccharide and Type III Secretion) | 2829 | 1.78 |
| PA3604 | response regulator ErdR | 654 | 1.77 |
| PA4117 | bacterial phytochrome, BphP | 2187 | 1.77 |
| PA3702 | probable two-component response regulator | 1044 | 1.75 |
| PA4032 | probable two-component response regulator | 717 | 1.71 |
| PA3878 | two-component sensor NarX | 1869 | 1.71 |
| PA4547 | two-component response regulator PilR | 1338 | 1.70 |
| PA1099 | two-component response regulator | 1422 | 1.69 |
| PA1180 | two-component sensor PhoQ | 1347 | 1.69 |
| PA2657 | probable two-component response regulator | 672 | 1.66 |
| PA4546 | two-component sensor PilS | 1593 | 1.66 |
| PA3204 | probable two-component response regulator | 678 | 1.65 |
| PA1098 | two-component sensor | 1209 | 1.62 |
| PA0928 | sensor/response regulator hybrid | 2778 | 1.61 |
| PA5124 | two-component sensor NtrB | 1077 | 1.59 |
| PA2583 | probable sensor/response regulator hybrid | 2979 | 1.57 |
| PA4381 | probable two-component response regulator | 684 | 1.54 |
| PA4725 | two-component sensor CbrA | 2952 | 1.54 |
| PA5360 | two-component response regulator PhoB | 690 | 1.53 |
| PA1336 | probable two-component sensor | 1902 | 1.51 |
| PA5166 | probable two-component response regulator | 1389 | 1.51 |
| PA3078 | probable two-component sensor | 1296 | 1.49 |
| PA3191 | probable two-component sensor | 1422 | 1.48 |
| PA5165 | probable two-component sensor | 1839 | 1.47 |
| PA5199 | AmgS | 1320 | 1.47 |
| PA3704 | probable chemotaxis sensor/effector fusion protein | 2310 | 1.47 |
| PA1798 | two-component sensor, ParS | 1287 | 1.46 |
| PA1157 | probable two-component response regulator | 711 | 1.45 |
| PA5483 | two-component response regulator AlgB | 1350 | 1.45 |
| PA1611 | probable sensor/response regulator hybrid | 1956 | 1.45 |
| PA5484 | KinB | 1788 | 1.44 |
| PA2523 | probable two-component response regulator | 675 | 1.43 |
| PA2656 | probable two-component sensor | 1338 | 1.42 |
| PA5125 | two-component response regulator NtrC | 1431 | 1.42 |
| PA0600 | probable two-component sensor | 2394 | 1.39 |
| PA4781 | cyclic di-GMP phosphodiesterase | 1182 | 1.38 |
| PA3346 | probable two-component response regulator | 1716 | 1.36 |
| PA0930 | two-component sensor | 1338 | 1.33 |
| PA1799 | two-component response regulator, ParR | 708 | 1.32 |
| PA5511 | MifR | 1344 | 1.32 |
| PA5361 | two-component sensor PhoR | 1332 | 1.31 |
| PA1437 | probable two-component response regulator | 690 | 1.30 |
| PA4398 | probable two-component sensor | 2097 | 1.29 |
| PA4102 | BfmS | 1305 | 1.26 |
| PA4380 | probable two-component sensor | 1281 | 1.25 |
| PA1243 | probable sensor/response regulator hybrid | 2577 | 1.25 |
| PA4885 | two-component response regulator | 690 | 1.20 |
| PA4983 | probable two-component response regulator | 735 | 1.19 |
| PA3271 | probable two-component sensor | 3480 | 1.17 |
| PA5512 | MifS | 1767 | 1.16 |
| PA4396 | probable two-component response regulator | 1101 | 1.15 |
| PA4776 | PmrA: two-component regulator system response regulator PmrA | 666 | 1.15 |
| PA2571 | probable two-component sensor | 1413 | 1.12 |
| PA0034 | probable two-component response regulator | 624 | 1.11 |
| PA4777 | sensor kinase PmrB | 1434 | 1.11 |
| PA3974 | Lost Adherence Sensor, LadS | 2388 | 1.11 |
| PA0464 | two-component sensor CreC | 1425 | 1.10 |
| PA1335 | probable two-component response regulator | 1278 | 1.10 |
| PA0756 | probable two-component response regulator | 672 | 1.10 |
| PA3077 | probable two-component response regulator | 672 | 1.09 |
| PA1396 | probable two-component sensor | 1623 | 1.08 |
| PA4112 | probable sensor/response regulator hybrid | 4254 | 1.08 |
| PA4101 | BfmR | 741 | 1.04 |
| PA2809 | two-component response regulator, CopR | 681 | 1.03 |
| PA4196 | BfiR | 645 | 1.02 |
| PA2479 | probable two-component response regulator | 681 | 1.01 |
| PA0757 | probable two-component sensor | 1383 | 0.97 |
| PA1397 | probable two-component response regulator | 633 | 0.97 |
| PA0463 | two-component response regulator CreB | 690 | 0.97 |
| PA3462 | probable sensor/response regulator hybrid | 2760 | 0.91 |
| PA2177 | probable sensor/response regulator hybrid | 2100 | 0.91 |
| PA3781 | probable transporter | 1281 | 0.91 |
| PA3947 | RocR | 1179 | 0.87 |
| PA0929 | two-component response regulator | 720 | 0.87 |
| PA1636 | two-component sensor KdpD | 2658 | 0.84 |
| PA1158 | probable two-component sensor | 1359 | 0.82 |
| PA1438 | probable two-component sensor | 1446 | 0.81 |
| PA4293 | two-component sensor PprA | 2769 | 0.80 |
| PA1637 | two-component response regulator KdpE | 693 | 0.80 |
| PA4036 | probable two-component sensor | 2301 | 0.77 |
| PA3946 | Two-component sensor RocS1 | 3639 | 0.75 |
| PA1992 | ErcS | 1695 | 0.72 |
| PA0471 | probable transmembrane sensor | 972 | 0.71 |
| PA4197 | BfiS | 2277 | 0.68 |
| PA3045 | Two-component response regulator, RocA2 | 624 | 0.62 |
| PA2810 | two-component sensor, CopS | 1332 | 0.52 |
| PA3714 | probable two-component response regulator | 642 | 0.43 |
| PA2480 | probable two-component sensor | 1323 | 0.40 |
| PA3044 | Two-component sensor RocS2 | 2226 | 0.36 |
| PA4886 | probable two-component sensor | 1392 | 0.35 |
| PA4982 | probable two-component sensor | 2997 | 0.26 |
| PA2882 | probable two-component sensor | 1116 | 0.26 |
| PA2524 | probable two-component sensor | 1419 | 0.21 |
| PA1976 | ErcS' | 2646 | 0.19 |
| PA1980 | response regulator EraR | 678 | 0.10 |
| PA2881 | probable two-component response regulator | 912 | 0.08 |
| PA1979 | sensor kinase, EraS | 651 | 0.00 |
