# Supplementary material for: Transcriptome profiling reveals links between ParS/ParR, MexEF-OprN, and quorum sensing in the regulation of adaptation and virulence in Pseudomonas aeruginosa
Source: BMC Genomics. 2013 Sep 13;14:618. doi: 10.1186/1471-2164-14-618 (PMC3848899; doi:10.1186/1471-2164-14-618)
Supplement: Additional file 7: Table S5 — Genes commonly regulated by ParS/ParR, MexEF-OprN and QS. [file 1471-2164-14-618-S7.doc]

| **Gene ID** | **Log Fold**  ***∆parS*/WT** | **Log Fold**  ***∆parR*/WT** | **Protein description** |  | | | | |
| --- | --- | --- | --- | --- | --- | --- | --- | --- |
| PA0007 | 1.37 | -0.89 | hypothetical protein |  |  |  |  |  |
| PA0059 | 1.69 | 2.40 | osmotically inducible protein OsmC |  |  | -2.11 | 0.66 | choline sulfatase, betC |
| PA0996 | 7.29 | 5.34 | probable coenzyme A ligase |  | | | | |
| PA0997 | 7.02 | 5.42 | PqsB |  | | | | |
| PA0998 | 5.90 | 4.22 | PqsC |  | | | | |
| PA0999 | 6.23 | 4.15 | 3-oxoacyl-[acyl-carrier-protein] synthase III |  | | | | |
| PA1000 | 4.97 | 2.75 | Quinolone signal response protein |  | | | | |
| PA1001 | 4.07 | 1.97 | anthranilate synthase component I |  | | | | |
| PA1002 | 5.90 | 3.80 | anthranilate synthase component II |  | | | | |
| PA1323 | 0.83 | 1.01 | hypothetical protein |  | | | | |
| PA1657 | 1.69 | 1.43 | conserved hypothetical protein |  | | | | |
| PA1658 | 1.67 | 1.16 | conserved hypothetical protein |  | | | | |
| PA1662 | 1.82 | 2.24 | probable ClpA/B-type protease |  | | | | |
| PA1664 | 3.23 | 3.61 | hypothetical protein |  | | | | |
| PA1667 | 1.58 | 2.02 | hypothetical protein |  | | | | |
| PA2067 | 3.60 | 1.08 | probable hydrolase |  | | | | |
| PA2068 | 4.12 | 0.86 | probable major facilitator transporter |  | | | | |
| PA2069 | 4.38 | 1.38 | probable carbamoyl transferase |  | | | | |
| PA2193 | 3.86 | 5.95 | hydrogen cyanide synthase HcnA |  | | | | |
| PA2195 | 0.79 | 2.82 | hydrogen cyanide synthase HcnC |  | | | | |
| PA2300 | 5.52 | 2.27 | chitinase |  | | | | |
| PA2433 | 3.14 | 2.49 | hypothetical protein |  | | | | |
| PA3326 | 2.80 | 3.33 | ClpP2 |  | | | | |
| PA3327 | 4.73 | 6.32 | probable non-ribosomal peptide synthetase |  | | | | |
| PA3329 | 6.66 | 6.82 | hypothetical protein |  | | | | |
| PA3330 | 5.50 | 5.34 | probable short chain dehydrogenase |  | | | | |
| PA3331 | 5.82 | 5.56 | cytochrome P450 |  | | | | |
| PA3332 | 7.12 | 6.61 | conserved hypothetical protein |  | | | | |
| PA3333 | 6.82 | 6.36 | 3-oxoacyl-[acyl-carrier-protein] synthase III |  | | | | |
| PA3334 | 6.75 | 6.58 | probable acyl carrier protein |  | | | | |
| PA3361 | 6.25 | 1.92 | fucose-binding lectin PA-IIL |  | | | | |
| PA3371 | 1.78 | 2.24 | hypothetical protein |  | | | | |
| PA3692 | 1.44 | 0.65 | Lipotoxon F, LptF |  | | | | |
| PA4132 | 1.43 | 2.62 | conserved hypothetical protein |  | | | | |
| PA4134 | 2.38 | 2.73 | hypothetical protein |  | | | | |
| PA4206 | 3.24 | 3.29 | RND efflux protein precursor MexH |  | | | | |
| PA4208 | 1.92 | 2.39 | outer membrane protein precursor opmD |  | | | | |
| PA4211 | 5.72 | 3.02 | probable phenazine biosynthesis protein |  | | | | |
| PA4738 | 1.17 | 0.97 | conserved hypothetical protein |  | | | | |
| PA4739 | 0.97 | 0.61 | conserved hypothetical protein |  | | | | |
| PA5220 | 4.22 | 3.94 | hypothetical protein |  | | | | |
