# Supplementary material for: Transcriptome profiling reveals links between ParS/ParR, MexEF-OprN, and quorum sensing in the regulation of adaptation and virulence in Pseudomonas aeruginosa
Source: BMC Genomics. 2013 Sep 13;14:618. doi: 10.1186/1471-2164-14-618 (PMC3848899; doi:10.1186/1471-2164-14-618)
Supplement: Additional file 8: Table S6 — Genes commonly regulated by ParS/ParR and MexEF-OprN, but not QS. [file 1471-2164-14-618-S8.doc]

| **Gene ID** | **Log Fold**  ***∆parS*/WT** | **Log Fold**  ***∆parR*/WT** | **Protein description** |  | | | | |
| --- | --- | --- | --- | --- | --- | --- | --- | --- |
| PA0283 | -0.59 | 5.00 | sulfate-binding protein precursor |  |  |  |  |  |
| PA0526 | 2.24 | 1.70 | hypothetical protein |  |  | -2.11 | 0.66 | choline sulfatase, betC |
| PA0958 | 1.62 | 2.79 | OprD precursor |  | | | | |
| PA1333 | -2.15 | -2.68 | hypothetical protein |  | | | | |
| PA1744 | -2.93 | -6.62 | hypothetical protein |  | | | | |
| PA1875 | 4.13 | 1.84 | probable outer membrane protein precursor |  | | | | |
| PA1970 | -7.41 | -8.96 | hypothetical protein |  | | | | |
| PA2486 | -4.99 | -4.83 | hypothetical protein |  | | | | |
| PA2491 | -3.51 | -2.39 | probable oxidoreductase |  | | | | |
| PA2493 | -8.21 | -7.30 | MexE |  | | | | |
| PA2494 | -8.75 | -9.36 | MexF |  | | | | |
| PA2495 | -7.11 | -8.53 | OprN |  | | | | |
| PA2624 | -0.20 | -0.99 | isocitrate dehydrogenase |  | | | | |
| PA2759 | -6.76 | -5.06 | hypothetical protein |  | | | | |
| PA2811 | -2.05 | -0.45 | probable permease of ABC-2 transporter |  | | | | |
| PA2812 | -2.62 | -0.86 | probable component of ABC transporter |  | | | | |
| PA2813 | -2.44 | -1.36 | probable glutathione S-transferase |  | | | | |
| PA3229 | -7.40 | -7.52 | hypothetical protein |  | | | | |
| PA3394 | 1.94 | -0.01 | NosF |  | | | | |
| PA3395 | 3.65 | 1.95 | NosY |  | | | | |
| PA3575 | -1.85 | -0.82 | hypothetical protein |  | | | | |
| PA3877 | 5.42 | 0.24 | nitrite extrusion protein 1 |  | | | | |
| PA3915 | 5.93 | 0.28 | molybdopterin biosynthetic protein B1 |  | | | | |
| PA4091 | 1.24 | 1.75 | 4-hydroxyphenylacetate 3-monooxygenase |  | | | | |
| PA4121 | -1.47 | 3.30 | conserved hypothetical protein |  | | | | |
| PA4124 | 0.44 | 2.02 | homoprotocatechuate 2,3-dioxygenase |  | | | | |
| PA4354 | -3.07 | -1.47 | conserved hypothetical protein |  | | | | |
| PA4356 | -3.55 | -2.94 | xenobiotic reductase |  | | | | |
| PA4623 | -4.55 | -4.91 | hypothetical protein |  | | | | |
| PA4661 | -1.01 | -0.72 | lipid A 3-O-deacylase |  | | | | |
| PA4881 | -10.68 | -9.13 | hypothetical protein |  | | | | |
| PA4918 | -2.37 | 0.41 | hypothetical protein |  | | | | |
| PA4919 | -1.48 | 0.20 | nicotinate phosphoribosyltransferase |  | | | | |
