# Supplementary material for: Transcriptome profiling reveals links between ParS/ParR, MexEF-OprN, and quorum sensing in the regulation of adaptation and virulence in Pseudomonas aeruginosa
Source: BMC Genomics. 2013 Sep 13;14:618. doi: 10.1186/1471-2164-14-618 (PMC3848899; doi:10.1186/1471-2164-14-618)
Supplement: Additional file 9: Table S7 — Genes commonly regulated by ParS/ParR and QS, but not MexEF-OprN. [file 1471-2164-14-618-S9.doc]

| **Gene ID** | **Log Fold**  ***∆parS*/WT** | **Log Fold**  ***∆parR*/WT** | **Protein description** |  | | | | |
| --- | --- | --- | --- | --- | --- | --- | --- | --- |
| PA0027 | 0.08 | 1.48 | hypothetical protein |  |  |  |  |  |
| PA0028 | 0.25 | 1.61 | hypothetical protein |  |  | -2.11 | 0.66 | choline sulfatase, betC |
| PA0050 | 1.69 | 0.66 | hypothetical protein |  | | | | |
| PA0105 | 1.59 | -0.60 | cytochrome c oxidase, subunit II, coxB |  | | | | |
| PA0108 | 1.83 | 0.75 | cytochrome c oxidase, subunit III, colll |  | | | | |
| PA0158 | 0.23 | 0.99 | TriC |  | | | | |
| PA0176 | 1.61 | -0.54 | aerotaxis transducer Aer2 |  | | | | |
| PA0855 | -0.23 | 1.02 | hypothetical protein |  | | | | |
| PA1130 | 3.07 | 2.42 | rhamnosyltransferase 2 |  | | | | |
| PA1131 | 3.12 | 1.10 | probable major facilitator transporter |  | | | | |
| PA1221 | 3.80 | 2.59 | hypothetical protein |  | | | | |
| PA1247 | 1.17 | 2.35 | alkaline protease secretion protein AprE |  | | | | |
| PA1249 | 2.18 | 0.77 | alkaline metalloproteinase precursor |  | | | | |
| PA1556 | -1.85 | 0.07 | Cytochrome c oxidase, CcoO subunit |  | | | | |
| PA1668 | 2.09 | 2.46 | hypothetical protein |  | | | | |
| PA1669 | 1.21 | 1.46 | hypothetical protein |  | | | | |
| PA1869 | 2.75 | 2.14 | probable acyl carrier protein |  | | | | |
| PA1870 | 4.24 | 3.42 | hypothetical protein |  | | | | |
| PA2076 | 0.79 | 1.25 | probable transcriptional regulator |  | | | | |
| PA2143 | 2.58 | 1.73 | hypothetical protein |  | | | | |
| PA2300 | 5.52 | 2.27 | chitinase |  | | | | |
| PA2303 | 0.92 | 1.53 | AmbD |  | | | | |
| PA2327 | 3.08 | 2.36 | probable permease of ABC transporter |  | | | | |
| PA2328 | 3.82 | 3.82 | hypothetical protein |  | | | | |
| PA2329 | 3.00 | 2.56 | probable ABC transporter |  | | | | |
| PA2331 | 2.55 | 2.25 | hypothetical protein |  | | | | |
| PA2338 | 1.44 | 2.57 | probable ABC maltose/mannitol transporter |  | | | | |
| PA2442 | 0.18 | -1.03 | glycine cleavage system protein T2 |  | | | | |
| PA2591 | 1.28 | 1.61 | probable transcriptional regulator |  | | | | |
| PA2592 | 2.15 | 1.20 | probable spermidine-binding protein |  | | | | |
| PA2593 | 3.18 | 2.53 | quorum threshold expression element, QteE |  | | | | |
| PA2939 | 2.93 | -0.10 | probable aminopeptidase |  | | | | |
| PA3022 | 2.12 | 2.86 | hypothetical protein |  | | | | |
| PA3205 | -2.01 | -5.16 | hypothetical protein |  | | | | |
| PA3335 | 4.88 | 4.43 | hypothetical protein |  | | | | |
| PA3346 | 1.28 | 0.59 | probable two-component response regulator |  | | | | |
| PA3476 | 2.04 | 1.98 | autoinducer synthesis protein RhlI |  | | | | |
| PA3477 | 1.29 | 1.15 | transcriptional regulator RhlR |  | | | | |
| PA3478 | 3.68 | 2.50 | rhamnosyltransferase chain B |  | | | | |
| PA3479 | 4.37 | 3.31 | rhamnosyltransferase chain A |  | | | | |
| PA3676 | 2.85 | 0.81 | RND efflux transporter |  | | | | |
| PA3677 | 3.03 | 0.81 | RND efflux membrane protein |  | | | | |
| PA3678 | 0.79 | -1.24 | probable transcriptional regulator |  | | | | |
| PA3724 | 4.29 | 3.32 | elastase LasB |  | | | | |
| PA3904 | 0.53 | 1.33 | hypothetical protein |  | | | | |
| PA4129 | 2.22 | 3.10 | hypothetical protein |  | | | | |
| PA4130 | 1.92 | 2.78 | probable sulfite or nitrite reductase |  | | | | |
| PA4131 | 1.51 | 2.68 | probable iron-sulfur protein |  | | | | |
| PA4172 | 2.03 | 2.00 | probable nuclease |  | | | | |
| PA4217 | 6.04 | 2.57 | flavin-containing monooxygenase |  | | | | |
| PA4442 | -0.42 | 2.69 | ATP sulfurylase |  | | | | |
| PA4443 | -0.40 | 2.73 | ATP sulfurylase small subunit |  | | | | |
| PA4677 | 0.32 | 1.03 | hypothetical protein |  | | | | |
| PA4691 | 0.32 | 1.21 | hypothetical protein |  | | | | |
| PA4916 | 1.19 | 1.30 | hypothetical protein |  | | | | |
| PA4917 | 1.65 | 1.54 | hypothetical protein |  | | | | |
