# Supplementary material for: Transcriptome profiling reveals links between ParS/ParR, MexEF-OprN, and quorum sensing in the regulation of adaptation and virulence in Pseudomonas aeruginosa
Source: BMC Genomics. 2013 Sep 13;14:618. doi: 10.1186/1471-2164-14-618 (PMC3848899; doi:10.1186/1471-2164-14-618)
Supplement: Additional file 10: Table S1 — Oligonucleotides used for gene cloning and qRT-PCR. [file 1471-2164-14-618-S10.doc]

| Oligonucleotide | Sequence (5’-3’) |
| --- | --- |
| par1 | GCTCAAGAGCGGAAGTGCTTTCAA |
| par2 | CCAGGTGTTCCATGTTTCGTGCAA |
| parSRT1 | ATCGAAAGCCTGATCAGCAA |
| parSRT2 | GCGCATGTCCTCTATCAGGT |
| parRRT1 | CGAGATCCTCAACCAGATCC |
| parRRT2 | GTTGAACAGGTAGCCCTTGC |
| lasIRT1 | CTACAGCCTGCAGAACGACA |
| lasIRT2 | ATCTGGGTCTTGGCATTGAG |
| lasRRT1 | GGACAGCCAGGACTACGAGA |
| lasRRT2 | ATGGACGGTTCCCAGAAAAT |
| rhlIRT1 | CTCTCTGAATCGCTGGAAGG |
| rhlIRT2 | TTTGCGGATGGTCGAACT |
| rhlRRT1 | GTTGCATGATCGAGTTGCTG |
| rhlRRT2 | CTCAGGATGATGGCGATTTC |
| pqsART1 | GGCTGAGTCCGGGTTACTG |
| pqsART2 | GGCCATTCACCTTGAACAGA |
| pqsCRT1 | CTGATCTGTTCGGCTTCCTC |
| pqsCRT2 | CAGCACTTTCTCGGCCTTAC |
| pqsDRT1 | CGAGTTCATCGTCGAACGTA |
| pqsDRT2 | GTGTTCACCAGCAACAGGTC |
| pqsRRT1 | CGTACTGCTCGACGATTTCA |
| pqsRRT2 | GAGCACGCACTGGTTGAAG |
| qteERT1 | TCAAGGAGTTCGACCTGCAC |
| qteERT2 | AACAGCGGAACGTGCTTTT |
| mexSRT1 | AATGTCATCCTCGACGAATTG |
| mexSRT2 | GAACTGCAGGTGCTTCTTGA |
| mexFRT1 | AGTACGCCGACATTCAGGAC |
| mexFRT2 | TGATGATGTTCTGGGTCTGC |
| oprNRT1 | CCTGGGTCTGTTCAGTCTGC |
| oprNRT2 | TCGAACTGTTTCCACCACAG |
| chiCRT1 | AACAACGACGCGATGAAAG |
| chiCRT2 | ATCGATCACGTAGCCGGTAG |
| phzA1RT1 | GCAGGGCTATTGCGAGAAC |
| phzA1RT2 | TCAAGTGGGAATACCGTCAC |
| phzA2RT1 | GCAGGGCTATTGCGAGAAC |
| phzA2RT2 | TCAGGTGGGAATACCGTCAC |
| phzMRT1 | AGTCCCGTTGCGTCTACGTC |
| PhzMRT2 | AGATCTCGAAGGCCACCAG |
| ropDRT1 | CGAGACGATCAACAAGCTCA |
| ropDRT2 | ATGGAGATCGGCTCTTTGG |
| rhlART1 | CCAGCAACCATCAGCACAT |
| rhlART2 | AGCTGCCGTTGATGAAATG |
| rhlBRT1 | TACGGGATTCCCTACCTGTC |
| rhlBRT2 | CAGCTTGAAGCGCTCGAT |
